# Development and management of gastrointestinal symptoms in long-term COVID-19

**DOI:** 10.3389/fmicb.2023.1278479

**Published:** 2023-12-14

**Authors:** Kai-Yue He, Xin-Yuan Lei, Lei Zhang, Dan-Hui Wu, Jun-Qi Li, Li-Yuan Lu, Umm E. Laila, Cui-Yun Cui, Zhi-Xiang Xu, Yong-Ping Jian

**Affiliations:** ^1^School of Life Sciences, Henan University, Kaifeng, China; ^2^Department of Blood Transfusion, Henan Provincial People’s Hospital, Zhengzhou, Henan, China

**Keywords:** long-term effects of COVID-19 (long-COVID), SARS-CoV-2, gastrointestinal symptoms, prognosis, therapeutics, gut microbiota, immune responses

## Abstract

**Background:**

Emerging evidence reveals that SARS-CoV-2 possesses the capability to disrupt the gastrointestinal (GI) homeostasis, resulting in the long-term symptoms such as loss of appetite, diarrhea, gastroesophageal reflux, and nausea. In the current review, we summarized recent reports regarding the long-term effects of COVID-19 (long COVID) on the gastrointestine.

**Objective:**

To provide a narrative review of abundant clinical evidence regarding the development and management of long-term GI symptoms in COVID-19 patients.

**Results:**

Long-term persistent digestive symptoms are exhibited in a majority of long-COVID patients. SARS-CoV-2 infection of intestinal epithelial cells, cytokine storm, gut dysbiosis, therapeutic drugs, psychological factors and exacerbation of primary underlying diseases lead to long-term GI symptoms in COVID-19 patients. Interventions like probiotics, prebiotics, fecal microbiota transplantation, and antibiotics are proved to be beneficial in preserving intestinal microecological homeostasis and alleviating GI symptoms.

**Conclusion:**

Timely diagnosis and treatment of GI symptoms in long-COVID patients hold great significance as they may contribute to the mitigation of severe conditions and ultimately lead to the improvement of outcomes of the patients.

## 1 Introduction

Coronavirus disease 2019 (COVID-19) caused by severe acute respiratory syndrome coronavirus 2 (SARS-CoV-2), is primarily recognized as an acute respiratory infectious disease (Zhu et al., 2020). SARS-CoV-2 has unique virological characteristics, associated with the rapid spread of SARS-CoV-2, which brings new challenges for the prevention and control of the long term outcomes of the epidemic (Hakim et al., 2021). Despite it remains challenging to determine exact number of individuals infected with COVID-19 over the last three or more years, evaluating the population affected by long-COVID is relatively easier to assess (Baroni et al., 2023). Long-COVID is defined as post-COVID-19 symptoms or post-acute sequelae of COVID-19 that involve illnesses associated with both direct and indirect impacts of SARS-CoV-2 infection (Levine, 2022). The World Health Organization defines “post-COVID-19 symptoms” as those of individuals with probable or confirmed SARS-CoV-2 infection who continue to have symptoms 3 months after infection, persisting for at least 2 months, and with no other obvious cause. The US Centers for Disease Control and Prevention uses a definition of persistent symptoms or health problems 4 weeks after infection, while the UK uses a 12-week time standard (Mansell et al., 2022; Soriano et al., 2022). In September 2022, leading data assessed that approximately 17 million people in Europe have been suffered from long-COVID and potentially millions may have to endure its effects for several consecutive years (Baroni et al., 2023). Three years after the onset of irresistible COVID-19, we have characterized major of its features, but we are still exploring about the potential prognosis and long-term outcomes of COVID-19 (Wang Z. et al., 2022).

Patients who have afflicted with COVID-19 for a prolonged duration predominantly experience persistent appearance of general influenza, including fever, cough, myalgia, and fatigue along with digestive manifestations such as abdominal pain, vomiting, anorexia, nausea, diarrhea, and elevated transaminase (Ren et al., 2021). Huang et al. (2020) also reported instances of “atypical” COVID-19 patients presenting with digestive symptoms as either their primary or sole manifestation (Chen N. et al., 2020). In addition, it has been reported that patients exhibiting digestive symptoms experience prolonged hospitalization and a less favorable prognosis compared with patients without gastrointestinal (GI) symptoms (Pan et al., 2020). Therefore, it is critical to investigate characteristics and underlying mechanisms of digestive system in long-COVID patients. In this review, we highlighted the prognosis and mechanism of intestinal symptoms with long-COVID more than 3 years since the outbreak onset, providing valuable and comprehensive evidence on the enduring effects of COVID-19. These endeavors aimed at monitoring the long-term outcomes of COVID-19 are critical for better understanding these clinical digestive manifestations and thus alleviating their consequences effectively.

## 2 Long-term GI effects of long-COVID

The spike protein of SARS-CoV-2 targets and attacks GI cells by specifically binding with the angiotensin-converting enzyme 2 receptor (ACE2) present on the surface of intestinal epithelial cells (Liang et al., 2020). Huang et al. (2020) revealed that 76% of hospitalized patients experienced at least one symptom that continues at least 6 months after infection of SARS-CoV-2, with intestinal manifestations being among those reported (Zhou et al., 2017; Cheung et al., 2020; Ong et al., 2020; Xiao et al., 2020; Joshee et al., 2022). Liang et al. (2020) identified the expression of ACE2 in various tissues showing that the small intestine bears the highest localization of ACE2, rendering IECs are more susceptible to SARS-CoV-2 infection (Carfì et al., 2020). SARS-CoV-2 is detected in feces of nearly half (48.1%) of COVID-19 patients (Cheung et al., 2020). A total of 70.3% of the patients with respiratory samples negative are notably stool samples positive for SARS-CoV-2 (Dhar and Mohanty, 2020; Pan et al., 2020; Xiao et al., 2020). The persistent existence of the SARS-CoV-2 genome in feces suggests that the virus continues to interact with the GI cells and cause prolonged clinical symptoms over time, with diarrhea being the primary manifestation (Zhou et al., 2017; Cheung et al., 2020; Ong et al., 2020; Xiao et al., 2020). Researchers detected the level of fecal SARS-CoV-2 RNA in mild-to-moderate COVID-19 patients 10 months after diagnosis and found that viral RNA shedding in feces was positively correlated with GI adverse conditions in patients with long COVID (Natarajan et al., 2022). A number of additional studies showed a similar result (Zollner et al., 2022; Upadhyay et al., 2023), suggesting there is an important biological significance for the continuous presence of SARS-CoV-2 in feces.

Zonulin (pre-Haptoglobin 2) is the precursor of haptoglobin (Hp)-2, whose uncleaved form detected in human serum is considered as a biomarker of increased gut permeability (Fasano, 2011). It has been reported that SARS-CoV-2 spike stimulates the expression of zonulin, leading to the increase of gut permeability (Llorens et al., 2021). Moreover, it was found that levels of zonulin in patients dying of severe COVID-19 are higher than those in patients recovered from the disease (Giron et al., 2021; Palomino-Kobayashi et al., 2022). These data suggest that zonulin might be associated with poor outcomes in COVID-19.

The majority of acute COVID-19-associated GI manifestations are mild and self-limiting and include anorexia, acute diarrhea, nausea, vomiting and abdominal pain/discomfort (Han et al., 2020; Pan et al., 2020). However, Weng et al. (2021) followed up 117 patients after Covid-19 for 90 days and found that patients with long COVID exhibit loss of appetite (24%), nausea (18%), acid reflux (18%), diarrhea (15%), and abdominal distension (14%) with < 10% of patients reporting belching, vomiting, and bloody stools. GI symptoms in long COVID are partially related to psycho-psychological factors, such as stress, anxiety, and depression, after recovery from COVID-19 (Freedberg and Chang, 2022). GI symptoms may not be evident during or prior of infection, but they can arise after a certain period or even manifest months after initial infection. Patients who have developed severe pneumonia followed by diminished blood oxygen saturation values are at higher risk of developing GI sequelae. This phenomenon could be attributed to the presence of multi-organ dysfunction syndrome that can exhibit in severe COVID-19 infection, particularly under the condition of septic shock (Luo et al., 2020; Wan et al., 2020). Notably, it is reported that GI manifestations during hospitalization for severe COVID-19 can lead to malnutrition, and this has been associated with raised mortality rates in patients (Zhang P. et al., 2021; Afrisham et al., 2023). Moreover, supplemental nutrition plays a pivotal role not only during hospitalization but also in alleviating GI sequelae effectively.

## 3 Mechanisms of long-term GI injury caused by long-COVID

### 3.1 SARS-CoV-2 infection of IECs

Intestinal organoid culture plays an important role in researches bridging the cell culture and *in vivo* animal work for disease models. SARS-CoV-2 is highly infectious in primates (including humans, rhesus monkeys, and crab eating monkeys), but has low transmission in wild-type mice, which greatly limits the research of SARS-CoV-2 in animals. SARS-CoV-2 is mainly thought to infect the lungs. In order to determine that SARS-CoV-2 can infect and replicate in the intestine, Zhou et al. (2020) established intestinal organoids from humans, a “mini-gut” cultured in a dish, and demonstrated that the virus can infect and replicate in intestinal cells of human intestinal organoids (Lamers et al., 2020), supporting the notion that the gut could be another crucial target organ for SARS-CoV-2. Moreover, researchers found that intestinal cells are susceptible to SARS-CoV and SARS-CoV-2 infection using human small intestinal organoids (hSIOs), and demonstrated that the intestinal epithelium supports SARS-CoV-2 replication (Lamers et al., 2020). Han et al. (2021) used intestinal organoids as a disease model for studying SARS-CoV-2 infection and screened inhibitors of SARS-CoV-2, providing valuable resources for drug screening and identifying candidates for COVID-19 therapeutics, arguing that intestinal organoids can indeed serve as an experimental model for coronavirus infection and therapeutic studies.

Multiple reports suggesting that viral nucleic acids are detected in anal/rectal swabs and stool samples from patients with mild and severe coronavirus pneumonia (Chen Y. et al., 2020; Liang et al., 2020; Lin et al., 2020; Wu et al., 2020; Xiao et al., 2020; Xu et al., 2020; Young et al., 2020), and the typical particles of SARS-CoV-2 are observed in IECs (Qian et al., 2021), indicating that SARS-CoV-2 actively replicates in the intestine. There are also studies suggesting that the presence of SARS-CoV-2 viral RNAs in GI tissue may be related to more severe cases (El Hajra Martínez et al., 2020). Massive infiltration of plasma cells and lymphocytes is observed in the lamina propria of the stomach, duodenum, and rectum, accompanied by the SARS-CoV-2 shell throughout the GI lumen, indicating that virus infection has triggered the inflammatory response in intestine (Liang et al., 2020; Xiao et al., 2020). Plasma VEGF level is markedly increased in patients with GI symptoms and positively correlated with intestinal inflammation. Mechanistically, Zeng et al. (2022) found that the spike of SARS-CoV-2 contributes to VEGF production in the duodenum of mice through activating the Ras-Raf-MEK-ERK pathway in IECs, but not in endothelium, and leading to an increase of permeability and inflammation. Moreover, epithelial-enteric neuronal crosstalk plays a vital role in SARS-CoV-2-induced inflammation. SARS-CoV-2 infection in enterocyte results in endoplasmic reticulum (ER) stress and the release of damage-associated molecular patterns (DAMPs) that induces the expression and release of vasoactive intestinal peptides (VIP) by enteric neurons (EN), hence disrupts gut electrolyte homeostasis. These findings highlight the role of epithelial-enteric neuronal crosstalk in COVID-19-related GI symptoms (Balasubramaniam et al., 2023). In addition, overexpression of interleukin (IL)-1β, IL-6 and TNF-α are also important in SARS-CoV-2-induced inflammation (Hoffmann et al., 2020; Villapol, 2020; Triana et al., 2021), supporting the notion that virus infection has triggered the inflammatory response in intestine.

**FIGURE 1:**
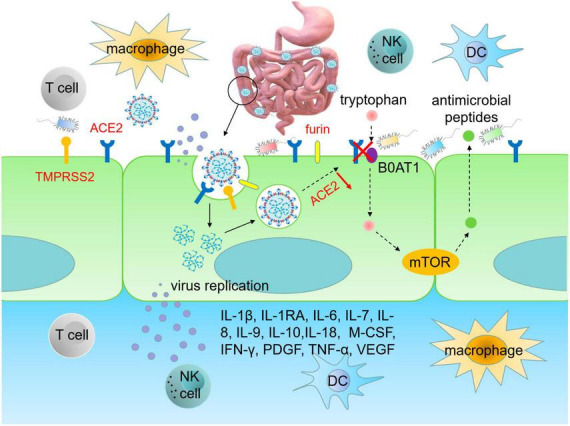
SARS-CoV-2 infects intestinal epithelial cells. The RBD region of S protein enhances the ability of SARS-CoV-2 to bind to ACE2 on intestinal cells. TMPRSS2 and furin promote RBD binding to ACE2 in IECs, thereby enhancing viral infection and causing GI damage. Upon infecting IECs, SARS-CoV-2 disrupts immune system, leading to an upsurge of pro-inflammatory cytokines, such as IL-1β, IL-1RA, IL-6, IL-7, IL-8, IL-9, IL-10, IL-18, M-CSF, IFN-γ, PDGF, TNF-α, and VEGF, which triggers cytokine storms. Infection of IECs by SARS-CoV-2 also leads to gut dysbiosis. The homeostasis of gut microbiota is related to the amino acid transport function of ACE2, which binds to the neutral amino acid transporter (B0AT1). Tryptophan, a neutral amino acid, regulates the expression of antimicrobial peptides, which maintain the flora of small intestine and large intestine in a stable state. RBD, receptor binding domain; ACE2, angiotensin converting enzyme 2; TMPRSS2, transmembrane serine protease 2; DC, dendritic cell; M-CSF, macrophage colony-stimulating factor; PDGF, platelet derived growth factor; VEGF, vascular endothelial growth factor.

Angiotensin-converting enzyme 2, as the main receptor for SARS-CoV-2 invasion, predominantly has higher expression and activity in the intestine. Therefore, the high expression of ACE2 in the gut is the key factor for SARS-CoV-2 to enter into host cells and induce GI symptoms (Du et al., 2020; Xiao et al., 2020; Zou et al., 2020). Structural biology and biochemical analysis reveal that SARS-CoV-2 is highly homologous to SARS-CoV, both belonging to β coronaviruses. The S protein of SARS-CoV-2 shares similarities with that in SARS-CoV, and the RBD structure of the SARS-CoV-2 S protein enhances its binding affinity to ACE2 on IECs. Proteins such as transmembrane serine protease 2 (TMPRSS2), endoprotease (Furin) and cathepsin L (CTSL) activate the S protein of SARS-CoV-2 to facilitate the fusion of SARS-CoV-2 and IECs membranes, thereby enhancing virus infection and leading to GI symptoms (D’Amico et al., 2020; Hoffmann et al., 2020; Lu et al., 2020; Wang M. Y. et al., 2020; Zhong et al., 2020). It can be inferred that SARS-CoV-2 adheres to ACE2-expressing cells and enters into IECs through receptor recognition, protease cleavage activation and membrane fusion, resulting in infiltration of plasma cells and lymphocytes and GI interstitial edema (Figure 1). The infected intestinal cells are thus damaged, leading to long-term malabsorption and intestinal inflammation.

### 3.2 Cytokine storm

Cytokine storm refers to the uncontrolled and excessive release of inflammatory factors due to immune dysregulation after stimulation. After SARS-CoV-2 infection, the immune system is disordered, resulting in cytokine storm (Chen N. et al., 2020). During conditions like COVID-19, homeostasis of intestinal microbiota is essential to maintenance of balanced immune reactions preventing an array of excessively detrimental inflammatory responses. The microbe-associated molecular patterns (MAMPs) of gram-negative bacteria activate inflammatory cells in COVID-19 conditions. Plasma levels of LPS produced by gram-negative bacteria are positively correlated with the severity of intestinal permeability in SARS-CoV-2 infection. The small intestine contains a significant amount of lymphoid tissues and numerous activated immune cells (Wang et al., 2023). Researchers have found the association of LPS with activated T cell and increased pro-inflammatory responses in the intestine (Clerbaux et al., 2022). Intestinal dysbiosis in some COVID-19 patients may contribute to the translocation of LPS from intestine into the portal circulation, hence stimulating the Kupffer cells in the liver and leading to the activation of NF-κB signaling and release of IFN-β and TNF-α (Li et al., 2015; Zuo et al., 2020b). When LPS enters into the systemic circulation, aforementioned response leads to an elevated hepatic and systemic inflammation (Kawaratani et al., 2013). Moreover, researchers found that low dose of LPS circulates in the plasma of patients with intestine dysbiosis and endotoxemia may act as a predictive cofactor in accelerating the severity of cytokine storm in COVID-19 patients (Szeto et al., 2008; Vignesh et al., 2020). In addition, IECs infected with SARS-CoV-2 release massive inflammatory mediators and chemokines, leading to the accumulation of neutrophils and further promoting the inflammatory response (Chen N. et al., 2020). When SARS-CoV-2 infects IECs, cytokines are excessively released, leading to long-term GI symptoms including predominantly diarrhea. According to Huang et al. (2020), levels of IL-1β, IL-1RA, IL-6, IL-7, IL-8, IL-9, IL-10, macrophage colony-stimulating factor (M-CSF), IFN-γ, platelet-derived growth factor (PDGF), TNF-α and vascular endothelial growth factor (VEGF) in COVID-19 patients were much higher as compared to healthy individuals, indicating that cytokine storm is being associated with the occurrence of extrapulmonary multiple organ dysfunction during the progression of COVID-19 (Effenberger et al., 2020; Ojetti et al., 2020).

Tao et al. (2021) followed 173 COVID-19 patients after post recovery and discharge and found that 52.3% of patients exhibit a mucosal immune response primarily driven by IgA, accompanied by increased serum creatinine, deteriorating proteinuria, and elevated levels of pro-inflammatory cytokines, notably IL-18 (Zhang P. et al., 2021). Plasma levels of IL-2, IL-7, IL-10, granulocytic stimulator, IP-10, human monocyte chemotactic protein 1, and human macrophage inflammatory protein 1α in ICU patients with COVID-19 are higher than those in mild patients, indicating strong correlation between cytokine storm and disease severity (Ma et al., 2020; Neurath, 2020; Au et al., 2021; Kumar et al., 2021). Virus-induced cytokine storm leads to rapid deterioration in COVID-19 patients with GI diseases, so immune-mediated cytokine storm plays a crucial role in the progression and development of COVID-19 (Figure 1).

### 3.3 Gut dysbiosis during long-COVID exposure

**FIGURE 2:**
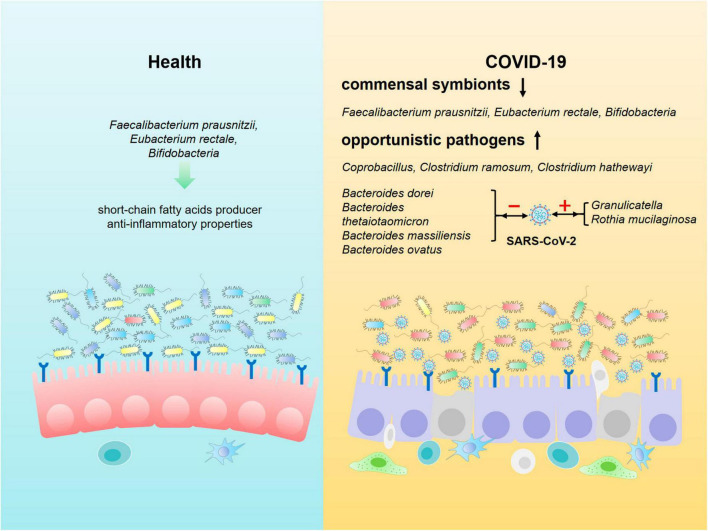
Composition of intestinal microbiota in healthy individuals and COVID-19 patients. Beneficial commensal bacteria such as *Faecalibacterium prausnitzii*, *Eubacterium rectale*, *Bifidobacteria* are dominant in the intestine of healthy population. However, the commensal symbionts are depleted, and opportunistic pathogens such as *Coprobacillus*, *Clostridium ramosum* and *Clostridium hathewayi* are markedly enriched in gut of COVID-19 patients. In addition, the composition of intestinal microbiota is closely associated with SARS-CoV-2 load in COVID-19 patients. *Bacteroides dorei*, *Bacteroides thetaiotaomicron*, *Bacteroides massiliensis* and *Bacteroides ovatus* are negatively correlated with the viral load, whereas the abundance of *Granulicatella* and *Rothia mucilaginosa* in oral and intestine of COVID-19 patients shows a positive correlation with SARS-CoV-2 load.

Researchers analyzed the composition of gut microbiota in feces from COVID-19 patients in acute, convalescent and post-discharge periods, and found that while the abundance of gut commensals in COVID-19 patients gradually increases as the patient showed clinical improvement and recovery, the profusion of flora remains significantly lower than that of healthy subjects at the three time points (Table 1). Even after the clearance of SARS-CoV-2 (confirmed by throat swabs) and resolution of respiratory symptoms, commensal depletion and gut dysbiosis persist and contribute to long-term (up to 30 days after clearance of SARS-CoV-2) GI complications (Zuo et al., 2020b; Yeoh et al., 2021). The baseline abundance of *Clostridium ramosum*, *Coprobacillus*, and *Clostridium hathewayi* is positively correlated with the severity of COVID-19 while disease severity inversely correlates with abundance of *Faecalibacterium prausnitzii, Eubacterium rectale* and *Bifidobacteria*, which possesses anti-inflammatory properties (Figure 2). *Bacteroides dorei, Bacteroides thetaiotaomicron, Bacteroides massiliensis*, and *Bacteroides ovatus*, which downregulate expression of ACE2 in murine intestine, are negatively correlated with SARS-CoV-2 load in fecal samples from COVID-19 patients during hospitalization. However, the abundance of *Granulicatella* and *Rothia mucilaginosa* in oral and intestinal samples of COVID-19 patients shows a positive correlation with the presence of SARS-CoV-2 (Zuo et al., 2020b; Gou et al., 2021; Wu et al., 2021; Yeoh et al., 2021). Tao et al. (2021) tracked and monitored 173 discharged COVID-19 patients, and observed that the level of virus-specific IgA antibody in serum of relapsed patients increases, while the diversity of gut microbiota decreases, but the abundance of conditional pathogens such as *Streptococcus* is amplified (Zhang P. et al., 2021). In addition, SARS-CoV-2 leads to dysbiosis of multiple microbiota. The abundance of bacteria, such as *Coprobacillus*, *Clostridium ramosum*, *Clostridium hathewayi*, *Erysipelotrichaceae*, *Actinomyces*, *Enterobacteriaceae*, *Parabacteroides*, *Alistipes_indistinctus*, *Fusobacterium*, *Streptococcus*, *Morganella*, *Neisseria*, *Burkholderia*, *Desulfovibrionaceae*, *Granulicatella*, and *Rothia mucilaginosa* is positively correlated with disease severity in COVID-19 patients (Table 2). Whereas the abundance of *Bifidobacterium*, *Dorea*, *Bacteroides*, *Anaerostipes*, *Lachnospiraceae*, *Roseburia*, *Alistipes_onderdonkii*, *Faecalibacterium*, *Blautia*, *Ruminococcus*, *Coprococcus*, *Eggerthella*, *Akkermansia*, and *Eubacterium rectale* is negatively correlated with disease severity in COVID-19 patients (Table 2).

**TABLE 1:** Alteration of gut microbiota in patients with acute Covid or long Covid.

	Acute Covid	References	Long Covid	References
Reduced relative abundance of gut microbiota	*Bifidobacterium adolescentis*	Yeoh et al., 2021; Venzon and Cadwell, 2022; Zhang et al., 2022	*Bifidobacterium adolescentis*	Yeoh et al., 2021; Venzon and Cadwell, 2022; Zhang et al., 2022
	*Ruminococcus bromii*	Schult et al., 2022; Venzon and Cadwell, 2022; Zhang et al., 2022	*Ruminococcus bromii*	Yeoh et al., 2021; Schult et al., 2022; Vestad et al., 2022; Zhang et al., 2022
	*Faecalibacterium prausnitzii*	Zuo et al., 2020b; Yeoh et al., 2021; Schult et al., 2022; Venzon and Cadwell, 2022; Zhang et al., 2022	*Faecalibacterium prausnitzii*	Tian et al., 2021; Yeoh et al., 2021
	*Fusicatenibacter*	Schult et al., 2022	*Eubacterium rectale*	Zuo et al., 2020b; Tian et al., 2021; Yeoh et al., 2021; Vestad et al., 2022
	*Blautia*	Schult et al., 2022	*Lachnospiraceae*	Vestad et al., 2022; Zhang et al., 2022
	*Lactobacillus*	Vodnar et al., 2020	*Erysipelotrichaceae*	Tian et al., 2021; Vestad et al., 2022
	*Lachnospiraceae bacterium*	Zuo et al., 2020b; Zhang et al., 2022	*Coprococcus*	Vestad et al., 2022
	*Eubacterium rectale*	Zuo et al., 2020b; Yeoh et al., 2021	*Barnesiella*	Vestad et al., 2022
	*Ruminococcus obeum*	Zuo et al., 2020b; Zhang et al., 2022	*Corynebacterium*	Vestad et al., 2022
	*Dorea formicigenerans*	Zuo et al., 2020b	*Subdoligranulum*	Vestad et al., 2022
	*Actinobacteria*	Yeoh et al., 2021	*Fusicatenibacter*	Vestad et al., 2022
	*/*	/	*Oscillospira*	Vestad et al., 2022
	*/*	/	*Clostridia*	Vestad et al., 2022
	*/*	/	*Bifidobacterium longum*	Yeoh et al., 2021
	*/*	/	*Collinsella*	Tian et al., 2021
	*/*	/	*Coriobacteriia*	Tian et al., 2021
Increased relative abundance of gut microbiota	*Bacteroides ovatus*	Zuo et al., 2020b; Yeoh et al., 2021; Zhang et al., 2022	*Bacteroides thetaiotaomicron*	Zuo et al., 2020b; Yeoh et al., 2021; Zhang et al., 2022
	*Bacteroides dorei*	Zuo et al., 2020b; Yeoh et al., 2021; Zhang et al., 2022	*Bacteroides caccae*	Zuo et al., 2020b; Yeoh et al., 2021; Zhang et al., 2022
	*Bacteroides thetaiotaomicron*	Zuo et al., 2020b; Yeoh et al., 2021; Zhang et al., 2022	*Agathobacter*	Newsome et al., 2021
	*Peptoniphilus*	Newsome et al., 2021	*Blautia*	Newsome et al., 2021
	*Corynebacterium*	Newsome et al., 2021	*Granulicatella*	Newsome et al., 2021
	*Campylobacter*	Newsome et al., 2021	*Klebsiella*	Newsome et al., 2021
	*Finegoldia*	Newsome et al., 2021	*Lactobacillus ruminis*	Yeoh et al., 2021
	*Comamonas*	Newsome et al., 2021	*Phocea*	Newsome et al., 2021
	*Sphaerochaeta*	Newsome et al., 2021	*Veillonella*	Vestad et al., 2022; Zhang et al., 2022
	*Synergistes*	Newsome et al., 2021	*Flavonifractor*	Tian et al., 2021; Vestad et al., 2022
	*Parabacteroides*	Schult et al., 2022	*Streptococcus*	Zhang et al., 2022
	*Veillonella*	Zhang et al., 2022	*Rothia*	Tian et al., 2021; Zhang et al., 2022
	*Coprobacillus*	Vodnar et al., 2020; Zuo et al., 2020b	*Erysipelatoclostridium*	Tian et al., 2021
	*Clostridium ramosum*	Vodnar et al., 2020; Zuo et al., 2020b	*Acidimicrobiia*	Tian et al., 2021
	*Clostridium hathewayi*	Vodnar et al., 2020; Zuo et al., 2020b	*Micrococcaceae*	Tian et al., 2021
	*Bacteroides massiliensis*	Zuo et al., 2020b; Yeoh et al., 2021	*Microtrichaceae*	Tian et al., 2021
	*Erysipelotrichaceae*	Zuo et al., 2020b	*Actinomyces*	Zhang et al., 2022
	*Streptococcus*	Zhang et al., 2022	*Bifidobacterium dentium*	Yeoh et al., 2021
	*Rothia*	Zhang et al., 2022	*Candidatus Microthrix*	Tian et al., 2021
	*Ruminococcus gnavus*	Yeoh et al., 2021	*Ruminococcus gnavus*	Liu et al., 2022
	*Ruminococcus torques*	Yeoh et al., 2021	*Bacteroides vulgatus*	Liu et al., 2022

**TABLE 2:** Composition of gut microbiota in COVID-19 patients.

Correlation type	Microbiota taxa	References
Positive correlation with COVID-19 severity	*Coprobacillus*	Zuo et al., 2020b; Najmi et al., 2022
	*Clostridium ramosum*	Zuo et al., 2020b; Najmi et al., 2022
	*Clostridium hathewayi*	Khan et al., 2021
	*Erysipelotrichaceae*	Li S. et al., 2021
	*Actinomyces*	Gu et al., 2020; Liu et al., 2022
	*Enterobacteriaceae*	Hughes et al., 2020; Amarsy et al., 2022; Marsico et al., 2022
	*Parabacteroides*	Al Bataineh et al., 2021; Farsi et al., 2022; Schult et al., 2022
	*Alistipes_indistinctus*	Zuo et al., 2020a
	*Fusobacterium*	Howell et al., 2021; Wolff et al., 2021
	*Streptococcus*	Amin-Chowdhury et al., 2021; Nair and Niederman, 2021; Parker et al., 2022
	*Morganella*	Zuo et al., 2021
	*Neisseria*	Rahaman et al., 2022; Romani et al., 2022
	*Burkholderia*	Zhong et al., 2021; Doubravská et al., 2022
	*Desulfovibrionaceae*	Sencio et al., 2022
	*Granulicatella*	Wu et al., 2021
	*Rothia mucilaginosa*	Marotz et al., 2021; Wu et al., 2021
Negative correlation with COVID-19 severity	*Bifidobacterium*	Zuo et al., 2020a; Ng S. C. et al., 2022; Romani et al., 2022
	*Dorea*	Zuo et al., 2020a
	*Bacteroides*	Prasad et al., 2022; Sun et al., 2022
	*Anaerostipes*	Romani et al., 2022
	*Lachnospiraceae*	Seibert et al., 2021; Sencio et al., 2022
	*Roseburia*	Ng S. C. et al., 2022
	*Alistipes_onderdonkii*	Zuo et al., 2021
	*Faecalibacterium*	Cortes et al., 2022; Sun et al., 2022
	*Blautia*	De Maio et al., 2021; Wang Y. et al., 2022
	*Ruminococcus*	Albrich et al., 2022
	*Coprococcus*	Romani et al., 2022; Xu et al., 2022
	*Eggerthella*	Romani et al., 2022
	*Akkermansia*	Cortes et al., 2022
	*Eubacterium rectale*	Tang et al., 2020; Yeoh et al., 2021

The homeostasis of gut microbiota is associated with the amino acid transport function of ACE2, which binds to the neutral amino acid transporter protein (B0AT1) on the luminal surface of IECs. Tryptophan, a neutral amino acid, is a key regulator of gut microbiota and inflammatory response. Tryptophan regulates the expression of antimicrobial peptides through the B0AT1-mTOR pathway to maintain the homeostasis of small intestinal and colonic flora (Hashimoto et al., 2012). After SARS-CoV-2 infects intestinal cells, IECs become dysfunctional, with some even necrotic and shedding, resulting in a reduction in the quantity of ACE2 (Trottein and Sokol, 2020; Verdecchia et al., 2020; Guo et al., 2021; Rajput et al., 2021). In addition, SARS-CoV-2 also co-internalizes with ACE2 through endocytosis, leading to a decreased expression of ACE2 and impaired transport of neutral amino acids, such as tryptophan, and inhibits the expression of antibacterial peptides, which contributes to gut dysbiosis and heightens vulnerability to intestinal inflammation (Figure 2). There is evidence showing that gut microbiota imbalance is related to changes in gut-lung axis (Dang and Marsland, 2019), gut-liver axis (Tripathi et al., 2018; Manzoor et al., 2022), and gut-brain axis (Mitrea et al., 2022) homeostasis in COVID-19 patients. Transmission electron microscopy analysis showed that the existence of SARS-CoV-2 particles is on the surface and inside of intestinal bacteria, suggesting that SARS-CoV-2 infection with human gut bacteria may be another mechanism leading to ecological imbalance in COVID-19 patients (Clerbaux et al., 2022). Moreover, SARS-CoV-2 infection in IECs triggers an immune response leading to gut dysbiosis, characterized by reduced levels of short-chain fatty acid produced by anti-inflammatory bacteria and an increase in the abundance of opportunistic pathogens, such as *Enterobacteriaceae* (Gu et al., 2020; Gaibani et al., 2021; Ren et al., 2021; Xu et al., 2021). Hence it is crucial to thoroughly assess the gut dysbiosis in long-COVID patients with diarrhea. If necessary, fecal metagenomic sequencing should be employed to identify novel therapeutic targets for the treatment of long-term GI pathogenesis by analyzing the characteristics of gut microbiota changes in COVID-19 patients.

### 3.4 Drug toxicity

Numerous anti-COVID-19 drugs have been undergone clinical trials. A combination of two or three drugs were often used for the treatment of COVID-19, which increased the risk of GI injury. It was reported that acute diarrhea was associated with the use of drugs such as oseltamivir and arbidol in 55.2% of COVID-19 patients (Ren et al., 2021). Furthermore, a study revealed that remdesivir, while used to combat COVID-19, may have adverse effects such as nausea, elevated ALT, and constipation (Panda et al., 2020), indicating that drug toxicity plays a critical role in the occurrence of GI injury among COVID-19 patients.

Moreover, antibiotics, non-steroidal anti-inflammatory drugs (NSAIDs) and corticosteroids are frequently employed in clinical settings to treat tissue hypoxia and cells injury caused by respiratory infection, including COVID-19. It was observed that many patients experience symptoms including nausea, abdominal pain and acute diarrhea following such treatment (Tian et al., 2020). In a study involving 1,099 patients with COVID-19, intravenous antibiotics were used in 57.5% of the patients, and among these patients 3.8% experienced acute diarrhea as an adverse effect (Guan and Zhong, 2020). By analysis of stool samples from 96 COVID-19 patients, Bernard-Raichon et al. (2022) concluded that opportunistic pathogens with antibiotic resistance massively proliferate and contribute to the aggravation of gut dysbiosis. Without administration of antibiotics, COVID-19 patients showed an enrichment of abundance in *Ruminococcus gnavus*, *Ruminococcus torques* and *Bacteroides dorei* and a reduction of *Bifidobacterium adolescentis*, *Faecalibacterium prausnitzii* and *Eubacterium rectale*. However, COVID-19 with antibiotic treatment was primarily related to an increase of *Parabacteroides*, *Sutterella wadsworthensis* and *Bacteroides caccae* and a decrease in *Adlercreutzia equolifaciens*, *Dorea formicigenerans* and *Clostridium leptum*. Administration of antibiotics during hospitalization was associated with the severity of COVID-19 (Yeoh et al., 2021). Therefore, digestive symptoms in COVID-19 patients could also be caused by the administration of anti-viral drugs, antibiotics, NSAIDs and corticosteroids. To mitigate long-term GI injury these drugs should be accompanied by protective agents as a combinational therapy.

### 3.5 Psychological effects of long-COVID

COVID-19 is a rapidly evolving infectious disease characterized by swift transmission, high pathogenicity, elevated mortality and lacks specific drugs for treatment. Consequently, most patients experience negative emotions including panic and anxiety. Stress-induced brain interactions stimulate GI tract and lead to abnormalities in digestive, sensory, and immune functions, resulting in GI symptoms such as abdominal pain and anorexia (Heinen et al., 2022; Taylor, 2022; Bassey et al., 2023). COVID-19 patients experience a wide range of physical and psychological symptoms, confirming that psychological factors can significantly slow down gastric emptying rates, that can ultimately lead to dysplasia (Aiyegbusi et al., 2021). A survey on the mental health and sleep status of COVID-19 patients found that compared with the control, the incidence of anxiety and sleep disorders are significantly higher in COVID-19 patients (Aiyegbusi et al., 2021), and the long-term GI symptoms in these patients has increased substantially (Anaya et al., 2021). Several studies suggest a causal impact of psychosocial stress on increased permeability of the intestine, possibly by corticotropin-releasing hormone-induced mast cell activation and reduced blood flow to the intestine under stimulation (Söderholm et al., 2002; Keita et al., 2010; Vicario et al., 2010). This suggests that psychological factors play a critical role in the development of GI symptoms in COVID-19 patients.

On the other hand, increased permeability of the intestine and abnormal influx of antigens from food and bacteria disrupts systemic immune homeostasis, which in turn harms brain function and structure (Genedi et al., 2019). Patients with structural and functional abnormalities in GI barrier, such as in colitis ulcerosa and Crohn’s disease, display a more frequently psychiatric comorbidity (Faresjö et al., 2007; Nicholl et al., 2008). Thus, GI symptoms mutually affect the outcome of psychological disorders.

### 3.6 Exacerbation of primary diseases

**FIGURE 3:**
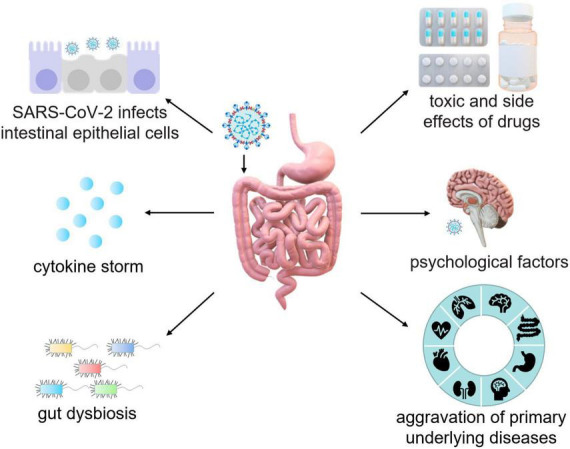
Schematic mechanisms of intestinal injury caused by SARS-CoV-2. The pathogenesis of intestinal injury in COVID-19 patients is mediated by the interaction between SARS-CoV-2 and ACE2, which leads to the destruction of epithelial cells and subsequent intestinal inflammation. In addition, of cytokine storm, gut dysbiosis, psychosocial factors, the aggravation of primary diseases, and potential side effects of antiviral drugs and antibiotics should also be considered during the treatment of GI injury in COVID-19 patients.

Patients with underlying diseases, such as chronic obstructive pulmonary disease, hypertension, coronary heart disease, diabetes, cerebrovascular disease, viral hepatitis B, and cancer are more likely to develop severe pneumonia and have poor prognosis from COVID-19 (Chen N. et al., 2020; Guan and Zhong, 2020). Patients with inflammatory bowel disease (IBD), including ulcerative colitis (UC) and Crohn’s disease (CD) are at high risk of developing COVID-19 complications, while long-term use of corticosteroids and immunosuppressants in IBD patients also elevates the risk of opportunistic SARS-CoV-2 infection (Bezzio et al., 2020). Moreover, IBD patients are more prone to stress and anxiety, which can lead to long-term GI symptoms (Figure 3).

## 4 Preventive measures for long-term GI injury induced by SARS-CoV-2

### 4.1 Probiotics alleviate SARS-CoV-2-induced long-term GI injury

Probiotics are considered as an effective intervention for reducing morbidity and mortality in severe COVID-19 patients (Finlay et al., 2021). Despite of the continuous emergence of novel SARS-CoV-2 variant strains, the most potent prevention and control measure remains the widespread vaccination of the population with protective vaccines (Lynn et al., 2022). Studies have shown that gut microbiota of pre-vaccinated individuals can influence the immune balance within the host, consequently impacting the vaccine efficacy and immunogenicity (Pulendran, 2019; de Jong et al., 2020). It was reported that probiotics could improve the vaccine immunogenicity, and increase the ratio of seroprotection and seroconversion in adults vaccinated with influenza vaccine (Lei et al., 2017). Thus, the effectiveness and immune response of the vaccine in vulnerable populations can be improved by increasing the abundance of probiotics. *Lactobacilli* in the intestine of COVID-19 patients are beneficial to the maintenance of gastrointestinal homeostasis and alleviation of inflammatory illnesses (Sun et al., 2018; Huang et al., 2020). Wang M. et al. (2020) applied *Lactobacillus plantarum* to express SARS-CoV-2 S protein and showed that the protein could be expressed on the surface of *Lactobacillus plantarum* and bears a high antigenicity for stimulating the generation of specific monoclonal and polyclonal antibody against SARS-Cov-2-S-protein. Thus, *Lactobacillus plantarum* recombined with the gene expressing SARS-CoV-2 S protein could be developed as an ideal oral vaccine for SARS-CoV-2 infection (Wang M. et al., 2020). In addition, probiotics downregulate the proportion of pro-inflammatory and anti-inflammatory cytokines, which is of great benefit to the alleviation of cytokine storms in patients with COVID-19 (Aziz et al., 2020; Li Q. et al., 2021). In a short, due to the role of probiotics in the regulation of innate immunity, preventive intake of probiotics (formulations) might be helpful to promote protective antiviral response and inhibit harmful excessive inflammatory response of host (Tomkinson et al., 2023).

Judicious use of probiotics and prebiotics can safeguard the intestinal barrier and reduce intestinal permeability, and hence alleviate intestinal symptoms in COVID-19 patients by regulating immune homeostasis and inflammatory response (Trompette et al., 2018; Mak et al., 2020; Hu et al., 2021). Emerging studies have shown that probiotics can increase the number and activity of T cells, which directly contribute to the immune response induced by T cells and enhances immune functions (Dhar and Mohanty, 2020). Furthermore, probiotics also enhance microbiome diversity and fortify the integrity of intestinal barrier functions, thereby effectively preventing microbial translocation (Mack et al., 2003; Reiff and Kelly, 2010; Wei et al., 2018; Suez et al., 2019; Badgeley et al., 2021). VSL#3, a mixture of 8 probiotics, has been safely and effectively used worldwide for decades. It is proven that VSL#3 alleviates enteritis and reinforces intestinal mucous barrier by improving the gut microecology (Mimura et al., 2004; Sartor, 2006; Tankou et al., 2018). Administration with VSL#3 is able help to improve the biodiversity of microbiota in patients, reduce fungal colonization, and increase the abundance of *Lactobacillus* and *Bifidobacterium*, thereby could diminish the severity of COVID-19-induced GI symptoms (Kühbacher et al., 2006; Chitapanarux et al., 2010). A comprehensive meta-analysis evaluated the clinical efficacy of probiotics in preventing treatment-related diarrhea (Wei et al., 2018), and showed that combined capsules containing diverse strains of *Bifidobacterium* and *Lactobacillus* were beneficial in reducing the incidence of acute diarrhea (Osterlund et al., 2007; Wada et al., 2010). Moreover, d’Ettorre et al. (2020) treated COVID-19 patients with a combination of *Streptococcus thermophilus DSM 32345*, *L. acidophilus DSM 32241*, *L. helveticus DSM 32242*, *Lacticaseibacillus paracasei DSM 32243*, *L. plantarum DSM 32244*, *LeviL. brevis DSM 27961*, *B. lactis DSM 32246*, and *B. lactis DSM 32247* for 7 days and found that patients given probiotics showed a reduction in GI symptoms as compared to those treated with the placebo. Gutiérrez-Castrellón et al. (2022) performed a randomized clinical trial involving 150 outpatients with COVID-19 treated with *L. plantarum KABP022*, *KABP023* and *KAPB033*, and *Pediococcus acidilactici KABP021* for 30 days and found that acute diarrhea remission rates are 53% and 28% in probiotic and placebo treatment groups, respectively. Similarly, Tang et al. (2021) applied probiotics to eliminate COVID-19 transmission in exposed household contacts involving 1,132 COVID-19 patients treated with *L. rhamnosus GG* or placebo for 28 days and beneficial effects were obtained. Collectively, current clinical evidence highlights that probiotics help to improve the immunity in COVID-19 patients by inhibiting pathogens colonization in the intestine, hence alleviating the disease severity (Olaimat et al., 2020).

Healthy dietary helps to maintain the homeostasis of gut microbiota in COVID-19 patients. Multiple studies showed that inflammatory response in COVID-19-positive patients is related to dietary styles (Ebrahimzadeh et al., 2022; Majidi et al., 2022; Moludi et al., 2022). Supplements of fruits, nuts, olive oil, vegetables, and whole grains, promote the abundance of probiotics and are negatively related to inflammation status in COVID-19 patients (Majidi et al., 2021; Hajipour et al., 2022). Moreover, vitamins D and A, selenium, flavonoids, zinc, and unsaturated fatty acids can alleviate inflammatory response in patients with COVID-19 via inhibiting the activation of nuclear factor kappa-B (NF-κB), hence reducing the production of pro-inflammatory cytokines, such as IL-6 and TNF-α. However, foods with higher glycemic load, carbohydrates, saturated fatty acids, and processed foods are found to disrupt the homeostasis of gut microbiota and are positively related to inflammation conditions in COVID-19 patients (Faghfouri et al., 2020, 2021; Zabetakis et al., 2020). In addition, appropriate intake of specific dietary fiber (Zhao et al., 2018), promote the growth of beneficial bacteria in the intestine, which may reduce the risk of SARS-CoV-2 infection (Kalantar-Zadeh et al., 2020; Merino et al., 2021). Fermentable dietary fiber (such as inulin) is fermented by gut microbiota to produce short-chain fatty acids (SCAFs), such as butyric acid, which alleviate the excessive inflammatory response caused by leukocytes in the lung, and enhance the immunoregulatory function of CD8 + T cells (Trompette et al., 2018). Antunes et al. (2019) administrated the acetate, butyrate, or propionate in the drinking water into mice infected with respiratory syncytial virus (RSV), and found that acetate exerts an anti-viral effect by binding with G protein coupled receptor 43 (GPR43). It suggests that increased abundance of probiotics could boost anti-viral ability and reduce long term GI symptoms in COVID-19 patients through preventive and therapeutic strategies.

### 4.2 FMT maintains intestinal flora homeostasis and relieves long-term GI symptoms caused by SARS-CoV-2

Fecal microbiota transplantation (FMT) is a therapeutic intervention to restructure the intestinal microbes of patients by transplanting the bacteria of healthy subjects into patient’s intestine (Xu et al., 2016; Cheng et al., 2018; Juul et al., 2018). Eiseman et al. (1958) treated pseudomembranous enteritis induced by antibiotics through FMT therapy, and obtained a notable improvement in patient condition, demonstrating the efficacy of FMT in treating Clostridium difficile infection and in the alleviation of intestinal inflammation (Hui et al., 2019). A significant proportion of COVID-19 patients bear gut dysbiosis, which indicates a close relationship between intestinal flora imbalance and SARS-CoV-2 (Cao et al., 2021). FMT treatment in severe COVID-19 patients, rapidly alleviates COVID-19 symptoms and cures Clostridium difficile infection (CDI) (Ianiro et al., 2020a; Biliński et al., 2022). Furthermore, Liu et al. (2021) observed that FMT improves the recovery of COVID-19 patients, alleviates residual gastrointestinal symptoms, and promotes the recovery of normal intestinal microbiota. Lastly, a retrospective study of 86 patients with CDI and COVID-19 showed that combination of antibiotics and FMT promotes the alleviation of abdominal pain and reduces relapse of CDI, and decreases levels of inflammation cytokines as compared to those treated with antibiotics alone (Boicean et al., 2022). Taken together, these studies suggest that FMT facilitates the relief of gastrointestinal symptoms, reduces intestinal inflammation, and promotes the recovery of COVID-19 patients.

Study in the germ-free SD rat model showed that the colonization of intestinal flora affects the expression of intestinal Ace2, Lcn2, and Nlrc5, and regulates systemic inflammatory responses, which impacts the susceptibility of IECs to SARS-CoV-2, indicating that normal gut microbiota may play a role in mitigating SARS-CoV-2 infectivity and resultant injury in gastrointestine (Yang et al., 2020). Therefore, selection of healthy subjects for FMT donor may prevent potential side effects transmitted from donor’s stool in gastrointestinal therapy (Ianiro et al., 2020b).

### 4.3 Rational medication for Long-COVID-19

COVID-19 patients may experience long term digestive symptoms due to the higher intake of antiviral drugs, antibiotics, NSAIDs and corticosteroids, with antibiotics being particularly associated with antibiotic-induced acute diarrhea (Ng T. M. et al., 2022). Despite the international community emphasis on reasonable antibacterial intervention for COVID-19 patients, clinical practice often deviates from this principle, resulting in an upsurge risk of developing antibiotic-induced acute diarrhea. Antibacterial treatment should be reserved for cases with clear evidence of bacterial infection (phlegm, procalcitonin, leukopenia and neutropenia) (Shchikota et al., 2021). Traditional Chinese medicine (TCM) has been reported to reduce the risk of mild and severe cases of COVID-19 developing to critical stages and significantly shorten the course of disease and improve overall clinical effectiveness (Liu et al., 2020). A large number of clinical trials tested the administration of TCM to treat COVID-19 and showed that some herbs could regulate the immune response, limit SARS-CoV-2 infection, and protect organs from viral infection-induced damage (Li et al., 2022). For example, Qiao et al. (2021) found that application of Qingfei Paidu decoction, Lianhua Qingwen Capsule, and Xuanfei Baidu formula could relieve the symptoms and reduce the production of pro-inflammatory cytokines in COVID-19 patients. Moreover, Li et al. reported that Lianhua Qingwen Capsule and Pudilan Xiaoyan Oral Liquid inhibit the expression of inflammatory factors, such as TNF-α, IL-6, CCL2/MCP-1, and CCL10/IP-10 (Deng et al., 2020; Runfeng et al., 2020). Polysaccharides are a kind of substances with active pharmacological activity and widely found in TCM. Polysaccharides play a crucial role in immunomodulatory, anti-fibrotic and antiviral functions. It was reported that polysaccharides alleviate the symptoms of COVID-19 by targeting the regulatory axis of transforming growth factor-β/Smad2/3 and DANCER/AUF-1/FOXO3 (Chen R. R. et al., 2020; Chen N. et al., 2020). In addition, polysaccharides that regulate homeostasis of intestinal flora for a long time by promoting the growth of probiotics (Xu et al., 2017), These findings suggest that TCM herbs and their active substances may be used in the prevention and treatment of COVID-19. Rational use of antibiotic or combination with TCM may be an effective way to reduce antibiotic related acute diarrhea in COVID-19.

### 4.4 Psychotherapy for overcoming post COVID-19 symptoms

COVID-19 patients often experienced concomitant psychological disorders like anxiety. Unfortunately, clinical focus tends to minimize physical symptoms, neglecting the accompanying mental and psychological problems that can contribute to the exacerbation of COVID-19 condition. The psychological state of COVID-19 patients has a significant impact on the development of the disease as an excessive psychological stress can lead to increased activation of GI tract, which increases the risk of dyspepsia and intestinal inflammation (Everitt et al., 2019). Psychotherapy served as a significant approach to enhance the quality of life and alleviate stress could shorten the course of COVID-19. In the realm of clinical care, medical staff should prioritize psychological care to ensure comprehensive wellbeing of patient.

## 5 Challenges and opportunities for long-COVID-19 treatment

Currently, there are multiple types of vaccine against SARS-CoV-2 for the prevention of the virus. However, there is no specific vaccination or authorized drug developed to directly treat COVID-19 (Majumder and Minko, 2021; Gasmi et al., 2023). The pathogenesis of long-term intestinal injury in COVID-19 patients is well-understood. Interaction between SARS-CoV-2 and ACE2 destroys epithelial cells leading to intestinal inflammation. Furthermore, during the treatment of COVID-19 patients it is critically needed to pay more attention toward underlying symptoms such as cytokine storm, gut dysbiosis, psychosocial factors, the aggravation of primary diseases, side effects of antiviral drugs and antibiotics (Figure 3). Therefore, clinicians must maintain heightened vigilance regarding digestive symptoms in COVID-19 patients. The expectation is for medical professionals to delve further into the comprehension of long-term symptoms in COVID-19 patients and better comprehend the pathogenesis of the disease, so as to provide more targeted treatment while effectively reducing the spread of the virus.

## 6 Conclusion

The pathogenesis of long-COVID is undoubtedly multifactorial. Even after the acute effects of COVID-19 subside, vigilant attention is required for an extended period to monitor the poor prognosis and sequelae of long-COVID. This review aims to comprehensively summarize and enhance the understanding of long-term GI injury caused by long-COVID, thereby improving the efficacy of clinical diagnosis and treatment, and paves a way for new therapeutic strategies and targets for GI injury.

## Author contributions

K-YH: Writing—original draft, Writing—review & editing. X-YL: Writing—original draft, Writing—review & editing. LZ: Writing—original draft. D-HW: Writing—original draft. J-QL: Writing—original draft. L-YL: Writing—original draft. UEL: Writing—original draft. C-YC: Writing—original draft. Y-PJ: Conceptualization, Funding acquisition, Writing—original draft, Writing—review & editing. Z-XX: Conceptualization, Funding acquisition, Writing—review & editing.
